# Double-bundle anterior cruciate ligament reconstruction improves tibial rotational instability: analysis of squatting motion using a 2D/3D registration technique

**DOI:** 10.1186/s13018-018-0825-y

**Published:** 2018-05-16

**Authors:** Kenichi Kidera, Akihiko Yonekura, Takeshi Miyaji, Yusuke Nakazoe, Kazuyoshi Gamada, Kei Yoneta, Futoshi Ikuta, Masato Tomita, Takashi Miyamoto, Shiro Kajiyama, Akira Hozumi, Ko Chiba, Narihiro Okazaki, Takayuki Shida, Makoto Osaki

**Affiliations:** 1 Department of Orthopaedic Surgery, Nagasaki University Graduate School of Biomedical Sciences, 1-7-1 Sakamoto, Nagasaki, 852-8501 Japan; 2 Medical Engineering and Technology, Graduate School of Medical Technology and Health Welfare Sciences, Hiroshima International University, Hiroshima, Japan

**Keywords:** Knee kinematics, Anterior cruciate ligament, Double-bundle anterior cruciate ligament reconstruction, 2D/3D registration technique

## Abstract

**Background:**

The anterior cruciate ligament-deficient (ACLD) knee requires appropriate treatment for the patient to return to sports. The purpose of this study was to clarify the kinematics of the anterior cruciate ligament-deficient knee in squatting motion before and after double-bundle anterior cruciate ligament reconstruction (DB-ACLR) using a 2D/3D registration technique.

**Methods:**

The subjects of this study were 10 men with confirmed unilateral ACL rupture who underwent DB-ACLR. Computed tomography (CT) of the knee joints was performed before DB-ACLR. Fluoroscopic imaging of the knee motion in squatting before and after DB-ACLR was also performed. The 2D/3D registration technique is a method of calculating positional relationships by projecting the 3D bone model created from the CT data onto the image extracted from the fluoroscopic images. The tibial anteroposterior (AP) and rotational positions were analyzed with reference to the femur.

**Results:**

The tibial AP position of the ACLD knees was significantly anterior to the contralateral knees (*p* = 0.015). The tibial rotational position of the ACLD knees was significantly internally rotated compared to the contralateral knees (*p* < 0.001). Both tibial AP and rotational positions improved after DB-ACLR (*p* < 0.001), with no significant differences compared to the contralateral knees.

**Conclusion:**

DB-ACLR improved not only tibial AP instability but also tibial rotational instability at knee flexion with weight-bearing. DB-ACLR appears to be a useful technique for normalizing the knee joint kinematics of ACLD knees.

## Background

Anterior cruciate ligament (ACL) tear is a common knee injury, with around 100,000 cases in the USA each year [1]. It has been reported that ligament failure after ACL rupture is a risk factor for knee osteoarthritis [1–3]. Abnormal kinematics of rotatory instability and anteroposterior (AP) instability are involved in the development of knee osteoarthritis (OA) in the ACL-deficient (ACLD) knee [4]. Load motion such as walking or crouching may cause arthropathy in such a state. Previous studies have reported that squatting in ACLD knees causes the tibia to move anteriorly and rotate internally [5, 6].

The ACLD knee requires appropriate treatment to prevent the onset and progression of knee arthropathy. In recent years, double-bundle ACL reconstruction (DB-ACLR) using hamstring tendon grafting has been reported to have good clinical outcomes and achieve static knee joint stability [7, 8]. However, the effects of DB-ACLR on kinematics in load flexion motion have not been fully clarified. The purpose of this study was to clarify the kinematics of the ACLD knee in squatting motion before and after DB-ACLR.

In order to demonstrate the effects of DB-ACLR, a 2D/3D registration technique [9, 10], which is less invasive than bone markers and more accurate than surface markers, was used [11, 12]. To verify the knee kinematics, fluoroscopic images of squatting movements were taken before and after DB-ACLR, and improvements of tibial AP and rotational movements were investigated. We wished to test the hypothesis that DB-ACLR improved the abnormal kinematics of squatting motions of ACLD knees. To test this hypothesis, before and after operative kinematics were measured in 10 DB-ACLR patients using 2D/3D techniques.

## Methods

### Subjects

This study was a cross-sectional study targeting patients with unilateral ACLD. This study was approved by the ethics committee of our facility. Patients who visited our hospital between 2009 and 2011 and who were diagnosed with ACL rupture were recruited. Ten ACLD patients participated in this study. The case number required was determined by G*Power ver. 3.1 software to complete the power analysis. The effect size was set to 0.33 with reference to our previous research. The sample size necessary for achieving alpha = 0.05 and beta = 0.20 was 8 in comparing the two dependent means.

The inclusion criteria included unilateral ACL rupture diagnosed by MRI. Male patients aged 20 years and over who understood the research contents participated in the research. Exclusion criteria were a history of trauma other than ACL injury, ACL rupture of the contralateral knee, malalignment of the lower extremity, and hip and ankle deformities.

All patients provided their written informed consent. The mean period from ACL injury to preoperative examination was 24.1 ± 37.3 months (range, 2 to 108 months). The mean age at the time of surgery was 29.6 ± 8.8 years (range, 20 to 47 years). The mean preoperative BMI was 23.69 ± 5.1 kg/m^2^. The Lachman test was positive in all cases, while the pivot-shift test was positive in eight cases. Based on the reports of Otsubo et al., DB-ACLR was performed using the ipsilateral hamstring tendon [13]. Lateral meniscus injury was confirmed in six knees, and medial meniscus injury was confirmed in four knees on intraoperative examination. Partial resections of three lateral menisci and three medial menisci were performed. The kinematic measurements were performed for the ACLD knees and the contralateral knees before and after DB-ACLR. Static AP stability was measured by a KT-2000 knee arthrometer (MEDmetric Corp., San Diego, CA, USA). The mean period from DB-ACLR to postoperative measurement was 24.6 ± 9.3 months (range, 12 to 37 months).

### Kinematic analysis

In vivo kinematics were analyzed using the 2D/3D registration technique proposed by Banks and Hodge [9]. The positional relationship of the femur and tibia is determined by projecting the 3D bone model created from the CT data onto the image extracted from the fluoroscopic image on the computer.

### Validity and reliability

Fregly et al. reported that the accuracy of this technique was 0.42 mm for in-plane translation and 1.3° for rotation [14]. Komistek et al. reported that the values were 0.45 mm for in-plane translation and 0.66° for rotation [15]. Moro-oka et al. reported that the accuracy of this technique was 0.53 mm for in-plane translation and 0.54° for rotation [16]. These studies indicated that this technique is accurate enough for evaluating knee kinematics of in-plane translation and rotation.

### Bone model creation and coordinate system embedding

Computed tomography (CT) (SOMATOM Definition, Siemens AG, Erlangen, Germany) of all knees was performed to create a 3D bone model. CT was performed with a 0.5-mm slice pitch spanning approximately 150 mm above and below the knee joint line. Then, 3D bone models of the femur and tibia were created from the CT images using 3D-Doctor (Able Software Corp., Lexington, MA, USA). The coordinate system for 3D bone models was 3D-Aligner (GLAB Corp., Higashi-Hiroshima, Japan).

**Fig. 1:**
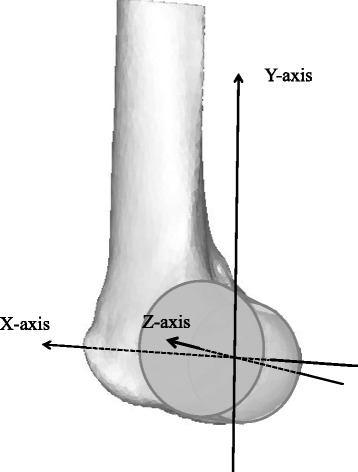
Coordinate system of the femur. The femoral condyles are regarded as a cylinder. *Z*-axis: the axis of the cylinder. *Y*-axis: the line through the origin parallel to the central line of the femoral shaft projected onto the sagittal plane. *X*-axis: the line perpendicular to the *Z*-axis and the *Y*-axis

The medial condyle and the lateral condyle of the femur were considered as one cylinder [17]. The central axis of the cylinder was set as the *Z*-axis of the femur. The origin was the midpoint of the central axis of the cylinder that penetrates between the medial and lateral bony surfaces of the femur. A plane through the origin perpendicular to the central axis was defined as the sagittal plane. The *X* and *Y* axes of the femur were set on this plane. The line passing through the origin of the femur and parallel to the central line of the projected femoral shaft to the sagittal plane was the *Y*-axis. The line passing through the origin and perpendicular to the *Z* and *Y* axes was defined as the *X*-axis (Fig. 1).

**Fig. 2:**
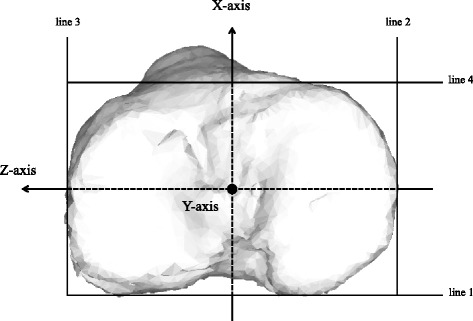
Coordinate system of the tibia. The tangent is set behind the tibial condyle (line 1) at the top level of the head of the fibula, and it is fitted onto the medial and lateral tangents perpendicular to the posterior tangent (lines 2 and 3), and the anterior tangent is set to create a rectangle (line 4). *Z*-axis: transverse bisector line of the rectangle. *X*-axis: anteroposterior bisector line of the rectangle. *Y*-axis: vertical line to the *X*-*Z* plane

The tangent was set posterior to the tibial condyle at the top level of the head of the fibula (Fig. 2, line 1). The tangents fitted onto the medial and lateral tibia perpendicular to the posterior tangent (Fig. 2, lines 2 and 3) and the anterior tangent (Fig. 2, line 4) were set to create a rectangle. The rectangle made from lines 1 to 4 was then translated to the tibial plateau. The midpoint of the rectangle was set as the origin of the tibia. The AP line of the bisector of the rectangle was set as the *X*-axis of the tibia, and the transverse bisector line of the rectangle and perpendicular to the *X*-axis was set as the *Z*-axis of the tibia. A line passing through the origin and perpendicular to the *X*-*Z* plane was set as the *Y*-axis of the tibia (Fig. 2).

### Squatting action

Squatting was performed by opening both legs wider than shoulder width, standing so that the left and right feet became 90° to each other, and bearing weight equally on both legs [18]. A squatting period was defined as the movement from the extended knee position to the maximum flexion knee position and returning to the extended knee position. Fluoroscopy was performed for the squatting period after it was practiced several times. The actual flexion knee angle of the subjects was about 100°, and the data up to 85°, to which all 10 cases could flex, were analyzed.

### Fluoroscopic imaging

The kinematics of the knee joint were examined using X-ray fluoroscopy with a square, 17-in., flat-panel screen (C-vision Safire, Shimadzu Corp., Kyoto, Japan). The imaging frame rate was 5 Hz, and the image size was 1024 × 1024 pixels. Still images were extracted from the kinematic data. The correction target was also projected on the same field of view of fluoroscopic images for distortion correction and optical calibration.

### Model registration

The bone model with the coordinate system setup was projected onto the distortion-corrected fluoroscopic image. The silhouette of the bone model was iteratively adjusted to match the silhouette on the fluoroscopic image with the custom Joint Track program (sourceforge.net/projects/jointtrack). Then, six degrees-of-freedom joint kinematics were computed using commercial software (3D-JointManager, GLAB Corp.). AP translation and rotation of the tibia referenced to the femur were measured. The kinematics were analyzed in 5° increments of knee flexion angles after B-spline curve approximation was performed.

### X-ray exposure dose

It was confirmed that the X-ray exposure doses of the subjects were 8 mSv with CT and 22 mSv with fluoroscopy. The fluoroscopic examination involved taking the three actions of squatting, kneeling, and knee extension. Only one fluoroscopic examination was performed, considering the exposure dose.

### Statistical analyses

Welch’s *t* test and the paired *t* test were performed using Statcel (OMS Ltd., Saitama, Japan), and post hoc pairwise comparisons were performed using a mixed linear model with repeated measures with SPSS version 22 (SPSS Inc., Chicago, IL, USA). The level of significance was set at *p* < 0.05.

## Results

**Fig. 3:**
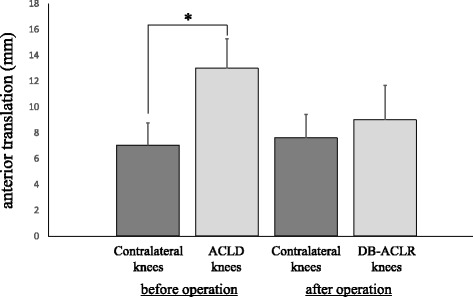
Anterior tibial translation of the ACLD and contralateral knees measured by KT-2000 knee arthrometer. Average magnitude of tibial anterior translation (mm). There is a significant difference between ACLD knees and contralateral knees before the surgery (Student’s *t* test, *p < 0.001)

The mean preoperative Lysholm knee scoring scale score was 79.3 ± 11.7, and after surgery, the mean was significantly improved to 98.9 ± 2.1 (*p* < 0.001). Anterior translation of the tibia was measured under anesthesia at DB-ACLR and at the time of the postoperative examination using KT-2000 (Fig. 3). Before DB-ACLR, the average anterior translation of ACLD knees was 13.0 ± 2.3 mm and that of contralateral knees was 7.1 ± 1.7 mm; there was a significant difference between the groups (*p* < 0.001). At the postoperative examination, the average of the DB-ACLR knees was 9.0 ± 2.7 mm, and the average of the contralateral knees was 7.6 ± 1.8 mm; there was no significant difference between the groups (*p* = 0.19). The average difference in the amount of anterior translation of the tibia between affected knees and contralateral knees was 6.0 ± 2.0 mm before the operation and 1.4 ± 2.4 mm after the operation; there was a significant difference (*p* < 0.001).

**Fig. 4:**
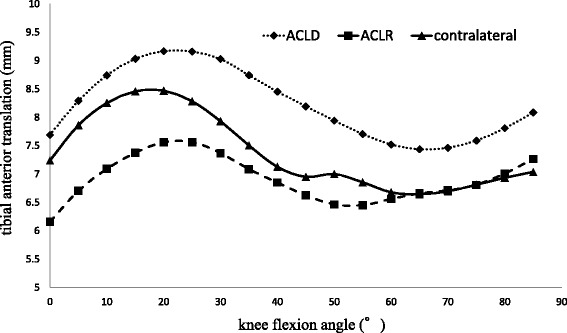
Anteroposterior translation of the tibia analyzed by 2D/3D registration technique. *Y*-axis: tibial anterior translation (mm). *X*-axis: knee flexion angle (°). Dotted line: ACLD knees. Dashed line: DB-ACLR knees. Solid line: contralateral knees. The anteroposterior position of the tibia of the ACLD knees is significantly different from the contralateral knees and the DB-ACLR knees (post hoc pairwise comparisons with a mixed linear model with repeated measures on SPSS, *p* = 0.015)

The mean magnitude of tibial AP translation (range minimum to maximum AP position) analyzed by the 2D/3D registration technique was 5.23 ± 2.70 mm in ACLD knees, 5.15 ± 3.84 mm in the contralateral knees, and 4.27 ± 2.34 mm in DB-ACLR knees; there were no significant differences. The AP position of the tibia of the ACLD knees was significantly different from that of the contralateral knees (*p* = 0.015) and the DB-ACLR knees (*p* < 0.001) on post hoc pairwise comparisons with a mixed linear model with repeated measures on SPSS. There was no significant difference in the AP position of the tibia between DB-ACLR knees and contralateral knees. The AP position of the tibia of ACLD knees was more anterior than that of contralateral knees at all flexion angles. The AP positions of the tibia of DB-ACLR knees were posterior to those of ACLD knees at all flexion angles. In addition, the AP positions of the tibia of DB-ACLR knees were more posterior to those of contralateral knees at 0°–60° of knee flexion, and they were almost the same at angles larger than 65° (Fig. 4).

**Fig. 5:**
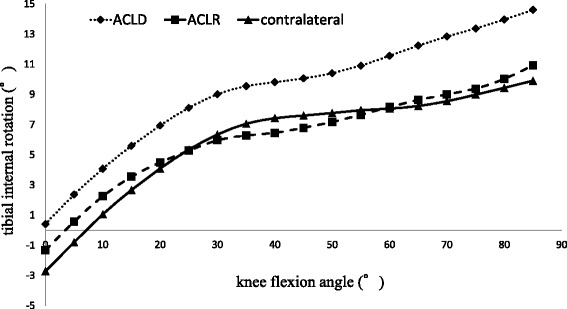
Rotation of the tibia analyzed by 2D/3D registration technique. *Y*-axis: tibial internal rotation (°). *X*-axis: knee flexion angle (°). Dotted line: ACLD knees. Dashed line: DB-ACLR knees. Solid line: contralateral knees. The rotational position of the tibia is significantly different between ACLD knees and contralateral knees, and between ACLD knees and DB-ACLR knees (post hoc pairwise comparisons with a mixed linear model with repeated measures on SPSS, *p* < 0.001)

The mean magnitude of the tibial rotation angle (range minimum to maximum rotational position) was 14.91° ± 6.64° in ACLD knees, 14.54° ± 5.51° in contralateral knees, and 12.87° ± 6.92° in DB-ACLR knees; there were no significant differences. The rotational position of the tibia of the ACLD knees was significantly different from that of the contralateral knees (*p* < 0.001) and the DB-ACLR knees (*p* < 0.001) on post hoc pairwise comparisons using a mixed linear model with repeated measures on SPSS. There was no significant difference in the rotational position of the tibia between DB-ACLR knees and contralateral knees. The tibial positions were more internally rotated in ACLD knees than in contralateral knees at all flexion angles. The tibial rotational positions of DB-ACLR knees and contralateral knees were almost the same at all flexion angles (Fig. 5).

## Discussion

In recent years, DB-ACLR using hamstring tendon grafting has been reported to produce good clinical outcome and joint stability [7, 8]. As a reason for the result, we hypothesized that DB-ACLR improved the abnormal kinematics of squatting motions of ACLD knees. To prove this hypothesis, before and after operative kinematics were measured in 10 DB-ACLR patients. We measured the kinematics using less invasive and more accurate 2D/3D registration technique [9–12]. DB-ACLR was found to control the AP translation of the tibia. Furthermore, DB-ACLR also controlled the internal rotation of the tibia. This is the first report to have analyzed the in vivo kinematics after DB-ACLR by comparison with the preoperative knee. The results of this study provide biomechanical support for the usefulness of DB-ACLR.

The 2D/3D registration technique was developed by Banks and Hodge [9]. The cardan angle was used to determine the three-dimensional positional relationship between the femur and tibia [19]. Co-author Gamada had worked with Banks using this method [20]. This method used single-plane fluoroscopic images. As mentioned before, this method is accurate for in-plane but less accurate for out-of-plane. Li et al. compared the ACLD knees and the contralateral knees using bi-plane fluoroscopic images. The results showed that the contact points of ACLD knees were significantly different from the contact points of the healthy side in the mediolateral direction, and ACLD knees had instability in the mediolateral direction [21]. Although bi-plane technique was more accurate, single-plane technique with a wide field of view seemed more convenient to analyze daily activities, such as wide-based squatting.

ACLD knees have rotatory and AP instability and have a risk of secondary damage [1, 3]. For example, Segawa et al. reported that plain radiographs of ACLD knees showed that 63% had OA, 37% of which had joint space narrowing [2]. von Porat et al. reported that radiographic changes were found in 78% of 122 ACLD knees, and of these, the radiographic OA equivalent to Kellgren and Lawrence grade 2 was seen in 41% [22]. There were also reports of abnormal kinematics of ACLD knees. Georgoulis et al. performed 3D optoelectronic gait analysis and reported a significant difference in tibial rotation angle during the initial swing phase in ACLD knees compared with ACL reconstructed and control knees [4]. DeFrate analyzed the forward lunge motion by the 2D/3D registration technique using the bone model constructed by MRI. They reported significant tibial anterior instability in ACLD knees at knee flexion angles of 0° and 15° [23]. They also reported significant tibial internal rotation at a knee flexion angle of 15°.

The squatting motion is one of the knee flexion movements with weight-bearing. It applies a heavy load to knee joints in activities of daily living. There have been a few reports of the kinematics of squatting motion in ACLD knees by the 2D/3D registration technique. Dennis et al. analyzed the medial and lateral condyle contact positions and femoral axial rotation in the squatting motion. The lateral condyle contact point shifted posteriorly, and the femoral axis rotated laterally for both normal knees and ACLD knees [5]. Yamaguchi et al. analyzed the relationship between the femoral and tibial axes. The ACLD knees showed greater tibial anterior translation from − 10° to 80° flexion and significant tibial internal rotation at full extension [6]. Chen et al. analyzed the medial and lateral condyle contact positions. The tibia was positioned significantly anterior at 15° flexion in ACLD knees [24]. Our previous research showed significant anterior translation and internal rotation of the tibia in ACLD knees [18]. In the present study, the same results were obtained.

Little has been reported on the kinematics after ACLR in loading motion [25–27]. Those reports mostly compared knee kinematics after ACLR with that of healthy control knees. To the best of our knowledge, only two reports by Isberg and Lin compared the same knees before and after ACLR. Isberg et al. analyzed medial and lateral femoral condyle translations and tibial rotation in active and weight-bearing knee extension movements by dynamic radiostereometric analysis (RSA) with tantalum markers. They evaluated patients preoperatively and followed them for 2 years after single-bundle anterior cruciate ligament reconstruction (SB-ACLR) using a four-strand ST/G autograft. The medial femoral condyle position after SB-ACLR was posterior to that of the intact knee in flexion angles from 0° to 25°, indicating that the knee was overstabilized in the AP direction. The lateral femoral condyle position was almost the same preoperatively and after ACLR. The tibial rotation kinematics nearly recovered to intact knee levels, but not significantly [11]. Lin et al. analyzed the medial and lateral condyle contact positions in a step-up motion by the 2D/3D registration technique using a bone model constructed by MRI. They evaluated patients preoperatively and followed them up at 6 and 36 months after SB-ACLR using a BTB autograft. There was no significant difference between before and 6 months after the operation. The medial condyle contact position was significantly anterior at 36 months after SB-ACLR. There was no significant change in the lateral condyle contact point among the three periods. They did not analyze tibial rotation [28].

We performed DB-ACLR, which is reported to be able to restore knee functions clinically [13, 29] and in a cadaveric study [30]. This is the first report that analyzed the in vivo kinematics after DB-ACLR by comparison with the preoperative knee. In the present results, the tibial position after DB-ACLR was posterior to the intact knee in flexion angles from 0° to 60°, which indicated that the knee was overstabilized in the AP direction. These results have almost the same meaning as the results by Isberg, who showed overstabilization in the AP direction in shallow flexion angles [11]. AP stabilization at shallow flexion angles is important in the treatment of ACL injury because the ACL injury often occurs at such flexion angles. Overstabilization at shallow flexion angles after ACLR may be reasonable for patients to return to sports, though long-term follow-up will be needed. On the other hand, in the present study, tibial rotation of DB-ACLR knees was almost the same as that of contralateral knees at all knee flexion angles. DB-ACLR could reconstruct the posterolateral bundle of the ACL, which is thought to play a more important role for knee joint rotational instability; thus, in the present study, tibial rotation was well controlled. However, Isberg et al. [11] and Ristanis et al. [31] reported that clinical outcomes were good even if tibial rotation was not improved. Longo et al. performed a systematic review of the papers comparing SB-ACLR and DB-ACLR [32, 33]. When comparing the clinical results of SB and DB, they reported that there was a statistically significant but no clinically significant difference in the results of KT arthrometer, and there was no statistically significant difference in the result of pivot-shift test. They recommended simple SB-ACLR until stronger evidence for DB-ACLR will be produced.

The limitations of this study are as follows. First, the subjects were only men. Although ACL tears occur frequently as noncontact-type injuries in women in their teens, there was concern about radiation exposure to young female participants, and only men were targeted in the study. However, female subjects are more likely to have hypermobility, which may alter the results. Second, since fluoroscopic imaging before and after surgery was performed once, the problem of reproducibility should be considered, but this protocol took into account the effects of radiation exposure. The subjects practiced the motions several times in order to take fluoroscopic images of stable motion. Third, these data were continuous data of static images. Thus, there was a possibility that they would be different from dynamic data. Fourth, subjects were not categorized according to the period from injury to survey or the state of the meniscus. These might have affected knee kinematics.

Based on the results of this study, DB-ACLR appears to be a useful technique for improving knee joint kinematics, especially for rotational instability. In the future, verification with non-load motion, deep flexing motion, etc., is also expected. It is significant that the usefulness of DB-ACLR was proven for load-flexing movement in activities of daily living. Based on this result, the long-term clinical results and the OA prevention effect of DB-ACLR are expected to be confirmed in the future.

## Conclusions

This is the first report to have analyzed the in vivo kinematics after DB-ACLR by comparison with the preoperative knee. Squatting motion was analyzed before and after DB-ACLR in ACLD knees using a 2D/3D registration technique. DB-ACLR improved not only tibial AP instability but also tibial rotational instability. DB-ACLR appears to be a useful technique for normalizing the knee joint kinematics of ACLD knees.

## Abbreviations

ACL: Anterior cruciate ligament
ACLD: Anterior cruciate ligament-deficient
AP: Anteroposterior
BTB: Bone-patellar tendon-bone
CT: Computed tomography
DB-ACLR: Double-bundle anterior cruciate ligament reconstruction
OA: Osteoarthritis
RSA: Radiostereometric analysis
SB-ACLR: Single-bundle anterior cruciate ligament reconstruction
ST/G: Semitendinosus/gracilis
